# Cloning, Transformation and Expression of Human Interferon α2b Gene in Tobacco Plant *(Nicotiana tabacum cv. xanthi)*

**Published:** 2012-08-25

**Authors:** Shahrzad Ahangarzadeh, Mohammad Hosein Daneshvar, Hamid Rajabi-Memari, Hamid Galehdari, Khalil Alamisaied

**Affiliations:** 1 Department of Agricultural Biotechnology, University of Agriculture and Natural Resources, Ahwaz, IR Iran; 2 Department of Cellular and Molecular Biology Research Center, Shahid Beheshti University of Medical Sciences, Tehran, IR Iran; 3 Department of Agronomy and Plant Breeding, Shahid Chamran University, Ahwaz, IR Iran; 4 Department of Genetics, Shahid Chamran University, Ahwaz, IR Iran

**Keywords:** Molecular Farming, Interferons, Tobacco, DNA Transformation Competence, Agrobacterium

## Abstract

**Background:**

Molecular farming is the production of important recombinant proteins in transgenic organisms on an agricultural scale. Interferons are proteins with antiviral and antitumor activities and can be used for viral infections and cancers treatments.

**Objectives:**

This study reports the transformation of INF α2b gene in tobacco plant for the first time in Iran.

**Materials and Methods:**

Interferon α2b gene was amplified by PCR using specific primers containing appropriate restriction enzymes, plant highly expression sequence and Histidine tag sequence. Target sequence was cloned in plant expression vector pCAMBIA1304 and the construct named pCAMINFα. pCAMINFα was transferred to E. coli strain DH5α and plated on LB agar medium containing kanamycin 50 mgl-1. The colonies were confirmed by colony PCR and sequencing. The construct was transferred into Agrobacterium tumefaciens by freeze-thaw method and transformed colonies were confirmed by colony PCR. Tobacco plants (cultivar xanthi) were inoculated with A. tumefaciens strain LBA4404 by leaf disc method. Inoculated explants were cultured on MSII (MS + BAP 1mgl-1 + NAA 0.1 mgl-1) at 28°C and darkness for 48 hours. Then explants were transferred to selection medium containing cephotaxime (250 mgl-1) and hygromycin (15 mgl-1) in a 16/8 (day/night) h photoperiod in growth room with an irradiance of 5000 lux. Transgenic plants were regenerated and transferred to perlite. Genomic DNA was extracted from regenerated plants by Dellaporta method at 5-leaf step and transgenic lines were confirmed by PCR with specific primers. Expression of Interferon α2b gene was confirmed by dot blotting.

**Conclusions:**

Since no report of interferon alpha production in plants in Iran has been expressed yet, this research could create a field of producing this drug in tobacco, in Iran.

## 1. Background

Molecular farming is the production of important recombinant proteins in transgenic organisms on an agricultural scale. While plants have been used as a source of medicinal compounds for so long, molecular farming represents a new source of molecular medicines, such as providing some important compounds like plasma proteins, enzymes, growth factors, vaccines and recombinant antibodies, whose medical benefits are well understood (1). production of foreign proteins in plants has numerous advantages including ease of genetic manipulations, efficiency of the transformation technology, speed of scale up, lack of potential contamination with human pathogens such as HIV, prions, hepatitis viruses, etc., conservation of eukaryotic cell machinery mediating protein modification and finally low cost of biomass production (2). Human IFN-α is an extracellular signal protein secreted by body cells when cells stimulated by viruses, microorganisms, foreign cells, foreign macromolecules, or various other chemical compounds (3-5). Then, IFN-α increases the defensive functions in their surrounding cells; these functions may regulate viruses replication, the immune responses, or cells growth (6, 7). Several cDNA clones of IFN-α have been isolated and expressed in prokaryotic cells and mammalian cells to prepare recombinant IFNα for clinical use (8, 9). Nowadays, clinical IFN preparations are used for anticancer and antiviral therapies (10).

The Food and Drug Administration first approved a particular subtype of interferon-α (IFN α2b) in 1986 for the treatment of hairy cell leukemia in the United States. The recombinant IFNα2b now on the market is produced using an E. coli expression system (2). Tobacco is a model plant system in molecular farming for last two decades. It has many advantages compared to other plants such as ease of gen transformation and tissue culture, high seeds number and huge amount of biomass (11), and the existence of many suitable expression vector for increasing of expression of interested genes. The high yield (one hundred tons per hectare of leaf product) and availability of different tobacco expression platform like plastid, transient and stable nuclear expression systems has made this plant as a good candidate for molecular farming research. Since this plant is not considered as food and feed crop it reduce the possibility of environmental contamination by tobacco transgenic lines (2). Transfer of foreign genes into higher plants mediated by Agrobacterium tumefaciens is a standard technique in plant molecular biology and genetic engineering (12). For many different plant species, it is possible to obtain transgenic plants after Agrobacterium mediated DNA transformation (13). Agrobacterium mediated transformation has been successfully exploited in many researches and increase the level of regeneration of normal plants (12).

## 2. Objectives

In this communication, we reported the transformation of INFα2b gene in tobacco and based on our knowledge, this work is the first research report of transformation of INFα2b gene in tobacco, in Iran.

## 3. Materials and Methods

### 3.1. Bacteria

Escherichia coli strain DH5α was used as a host for maintaining and proliferating the construct and Agrobacterium tumefaciens strain LBA4404 for transformation of the INF α2b gene to tobacco. The bacteria were provided by Dr. Mokhtar Jalali from Tarbiat Modarres University, Tehran, Iran.

### 3.2. Primers

The appropriate primers were designed considering plant high expression sequence (Kozak sequence), INFα2b gene both sides, adequate restriction sites, His Tag sequence to detect expression and purification and factor Xa for removing His Tag sequence. These specific primers were used for amplification of this gene. The nucleotide sequences of the primers were as follow: Forward Primer:

5´CATGCCATGGCACATCATCATCATCATCATCAACAATGTGATCTGCCTCAAACC-3′ contained NcoI recognition site and reverse primer: 5´CATCAGGGTCACCCTATTATT TCCTTATTCTTAAACTTTC-3´contained recognition site for BstEII.

Vector:

Plant expression vector pCAMBIA1304 (CAMBIA Co. Australia) was used which carries kanamycin-resistance gene for selection of the colonies of the bacteria and hygromycin-resistance gene for the transgenic lines. This vector carries LB and RB (Left and Right Borders for integration of foreign genes into host genome, CaMV35s, (Cauliflower Mosaic Virus promoter which induces high level of transcription), NcoI and BstEII restriction sites and NOS, (Nopaline Synthase terminator which induce termination process rate) GFP and GUS gene (As reporter genes).

### 3.3. Plant Materials

Tobacco (Nicotiana tabacum cv. xanthi) was used. Young leaves were selected for tansformation. This cultivar (xanthi) is a model for gene transformation studies and is not used for cultivation (provided from Agricultural Biotechnology Laboratory of Tarbiat Modarres University, Tehran, Iran).

### 3.4. Cloning of INFα-2b Gene in pCAMBIA1304

**Figure 1:**
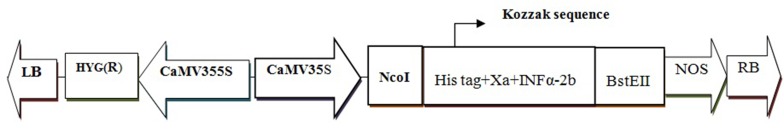
T-DNA Region of pCAMINFα-2b. LB and RB: Left and Right Borders, HYG(R): Hygromycin Selectable Marker, CaMV35s: Cauliflower Mosaic Virus Promoter, NcoI and BstEII: Restriction Sites and NOS: Nopaline Synthase Terminator.

The INFα2b gene has been already cloned in the pALCA vector. This vector was used as a template for PCR. The gene was amplified using mentioned primers, and then, PCR product was extracted from the gel using QIAGEN kit. So, this was digested with BstEII and NcoI (NEB Co). The digested PCR product was extracted from gel using gel extraction kit again and then ligated with digested pCAMBIA1304 vector by T4 DNA ligase in 16°C overnight and the results were transformed to E. coli. Consequently, the INFα2b fragment was replaced in GUS- GFP region of pCAMBIA1304 under the control of the CaMV35s promoter and the NOS terminator by ligation process (Figure 1).

### 3.5. Transformation of E. coli Using Heat Shock Method

**Figure 2:**
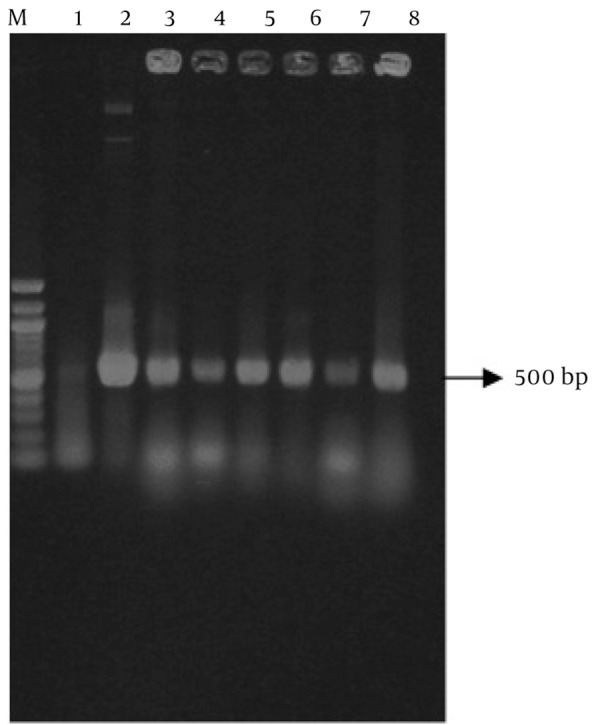
Confirmation of Cloning of INFα-2b Gene in E. coli by Colony PCR Technique. M: GeneRuler™ 100 bp DNA Ladder (Fermentase), Lane1: E. coli Without pCAMBIA-INFα (Negative Control), Lane 2: Positive Control (pCAMIFNα), Lane 3-8: Random Colonies Selection.

For transformation of recombinant vector to E. coli, 5µl of pCAMINFα binary vector was mixed with 1.5 ml of competent cells of E. coli strain DH5α and then transferred in a microtube and was put on ice for 30 min. Vector was immediately submerged in bain-marie for 90 seconds at 42°C and then, was put on ice for 2 min. 800 µl of LB (Luria-Bertani Broth) was added to microtube. The culture was grown for one hour at 37°C on shaker. Eventually, 100µl of transformed cells were cultured on the LB plates containing 50 mg/L kanamycin. Plates were incubated for 16-18 hours at 37°C (14). The pCAMINFα recombinant plasmid was verified by colony PCR (Figure 2), digestion and sequencing.

### 3.6. Transformation of A. tumefaciens Using Thaw and Freezing Method

**Figure 3:**
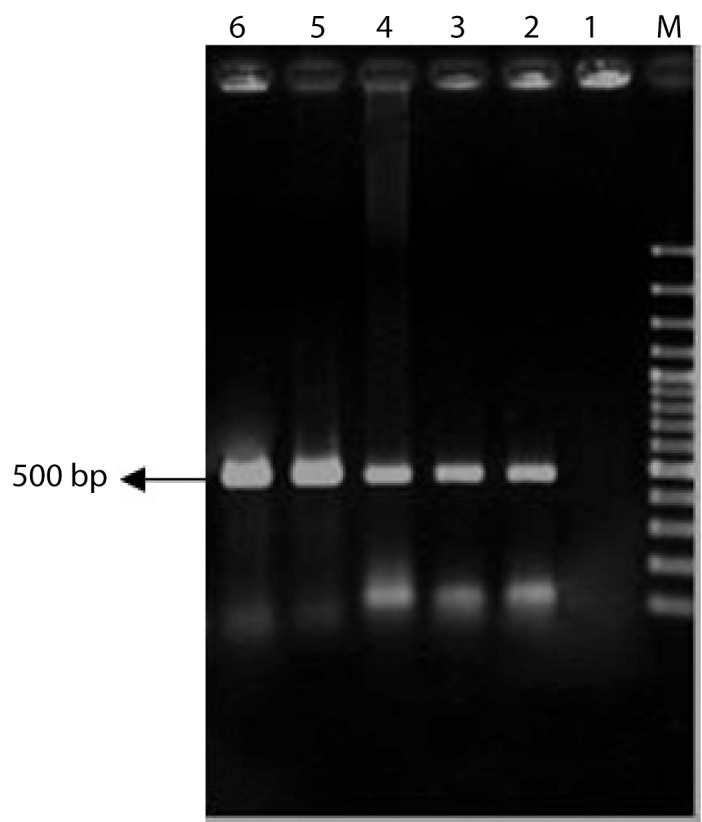
Confimation of INFα-2b Gene in Agrobacterium by Colony PCR Technique. M: GeneRuler™ 100 bp DNA Ladder (Fermentase), Lane1: Agrobacterium Without pCAMBIA-INFα (Negative Control), Lane 2: Positive Control (pCAMIFNα), Lane 3-6: Random Colonies Selection.

A. tumefaciens strain LBA4404 was grown overnight at 28°C in liquid LB medium with 80mg/L streptomycin. Agrobacterium culture spun down at 3500 rpm for 10 min and resuspended in 100 µl of 20 mM CaCl2. 5 µl of vectors were added to suspension and mixed culture was submerged in liquid nitrogen and then in bain-marie for 5 min at 37°C. One ml of LB was added and culture was grown for 3 hours in a 28°C shaker. 100 µl of transformed cells were cultured on the LB plates with a combination of 50 µg/ml kanamycin and 80 mg/L streptomycin. Plates were incubated for two days at 28°C (14) (Figure 3).

### 3.7. Plant Transformation

Tobacco plants were grown on MS medium (15) and the young leaves (length up to 4 cm) were used for transformation of the Agrobacteria suspension (with OD600 nm = 1.0). The leaves were cut into 8-10 pieces and transferred to petri dishes containing MS II medium (MS medium supplemented with BAP and NAA) and incubated at 26°C in the dark for two days. Leaf pieces were transferred to MS III (MS II + 15 mg/lit hygromycin + 200 mg/lit of cephatoxim) plates and incubated at 25°C with a 16/8 (day/night) h photoperiod for three to four weeks. The shoots were removed and transferred onto MS IV (MS III without NAA + BAP) and incubated at 25°C with a 16/8 (day/night) h photoperiod for 14 days until root was developed. The small plants were transferred into jar glasses containing MS IV and incubated at 25°C in a 16/8 h (day/night) light rhythm for two weeks. The tobacco plants were transferred to vase including perlite and were grown for two to three weeks. Finally, the plants were transferred to soil.

### 3.8. Polymerase Chain Reaction (PCR) Analysis of Transgenic Plant

Genomic DNA of transgenic plants was extracted, using Dellaporta method (16). PCR amplification of genomic DNA in order to detect presence of INFα-2b gene was carried out using primers described above, so plants with INFα-2b gene were specified.

### 3.9. Immunoblot Analysis

Total soluble proteins were extracted from whole leaves. Dot blot analysis was performed on wild type and transgenic lines. Solution preparation and its methods were described by Roche Company which provides Anti-His6Peroxidase. 10 ng protein samples were dotted on nitrocellulose membrane sheets. Then, western blocking solution 1X was added and incubated for one hour at 20°C. Solution was washed out and replaced with Anti-His6Peroxidase. It was incubated for 90 min, and then after the solution was poured out, washing with TBST1x was repeated three times for 5 min each time. Finally antibody and substrate were added. After colour development, the membranes were washed out with water and the colour changing was evaluated in both transgenic and control plants.

## 4. Results

### 4.1. Plant Transformation

**Figure 4:**
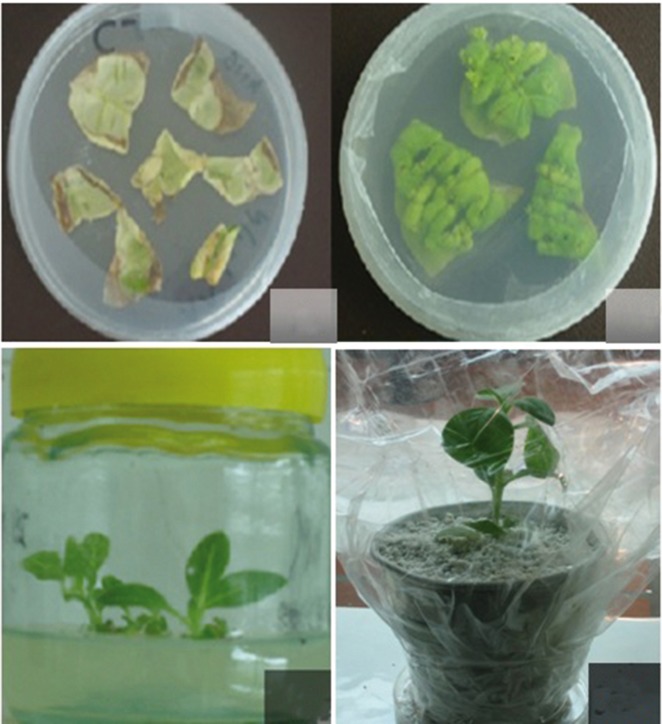
Regeneration of Transgenic Tobacco (Nicotiana tabacum cultivar xanthi) Plants. Leaves Were Inoculated by Agrobacterium tumefaciens, without pCAMINFα as Control (A) and With pCAMINFα (B) on MSIII Medium (MS II + 15 mg/lit Hygromycin + 200 mg/lit Cephatoxim). Regeneration and Rooting on MSIV Medium (MS III Without Hormone) (C), Transporting to Perlite (D).

Tobacco plants (cultivar xanthi) were inoculated with A. tumefaciens strain LBA4404 by leaf disc method. Inoculated explants were cultured on MSII (MS+ BAP 1mgl-1 + NAA 0.1 mgl-1) at 28°C and darkness for 48 hours. Then explants were transferred to selection medium containing cephotaxime (250 mgl-1) and hygromycin (15 mgl-1) in a 16 h light and 8 h darkness rhythm in growth chamber. Transgenic plants were regenerated and transferred to soil (Figure 4). 

### 4.2. Extraction of Genomic DNA and PCR Analysis

**Figure 5:**
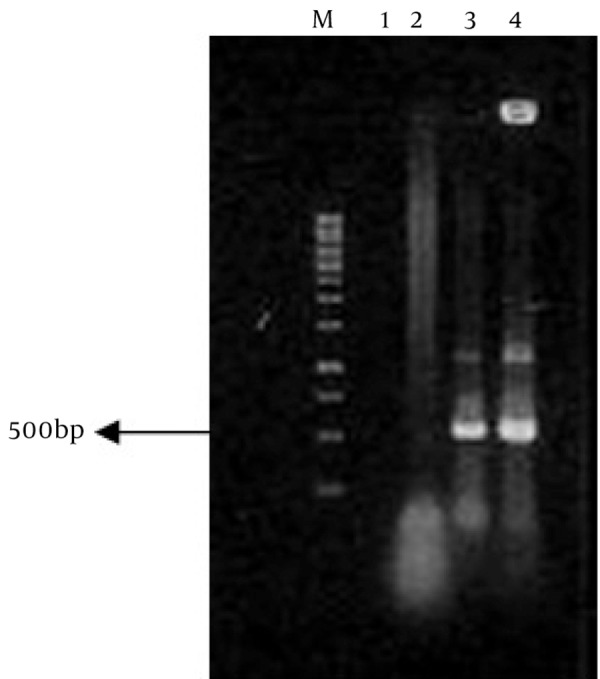
PCR Analysis of Transgenic Plants. M: 1kb Marker, Lane 1: ddH2O, Lane2: Wild Type Plant, Lane 3: Transgenic Plant, Lane 4: Positive Control (pCAMIFNα).

Genomic DNA of transgenic and control plants were extracted using Dellaporta method. The presence of INFα-2b gene in transgenic plant was proofed and detected using PCR technique, while control plants did not show any band on agarose gel electrophoresis (Figure 5).

### 4.3. Protein Extraction and Confirmation of Gene Expression

**Figure 6:**
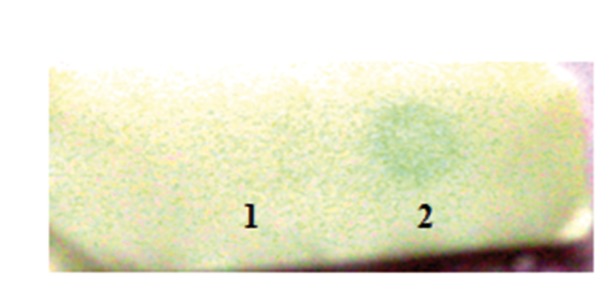
Dot Blot Analysis of Protein Extraction of Tobacco. Lane 1: Protein Extraction of Negative Control Plants (Wild Type), Line 2: Protein Extraction of Transgenic Plant.

The total extracted soluble proteins from both transgenic and wild type plants were used for immunedot blotting. The Figure 6 shows the results of the dot blot assay which confirms gene expression in transgenic plant. Screened transgenic lines as well as wild type plants were used for immunodot blot analysis. The expression of human interferon α2b which has His tag in the C-terminal can be detected by anti His tag peroxidase and turn the substrate colour while the extracted protein from wild type plant did not show any turning colour in dot blot assay.

## 5. Discussion

Traditional production systems that use bacterial fermentation, insects and mammalian cell cultures, and transgenic animals have undergone drawbacks because of some reasons include; high expenses, scalability, product safety and authenticity (1). Producing of biopharmaceuticals in plants has some advantages compared to other prokaryotic and eukaryotic systems. So the use of plant expression platform as host for recombinant protein production has become more popular in research and development of new medicines (17). Both transient and stable expressions have been used for recombinant proteins expression in plants. The stable method was used in our study. Although the time for generating stable transgenic plants is more than transient expression however the transgenic tobacco plants can generate millions of seeds which can produce high quantity of recombinant proteins in next steps. In the present study, the binary vector, pCAMBIA1304 was used in plant transformation. This vector carries CaMV35S promoter and NOS terminator. In dicot plants, CaMV35S is a suitable promoter because it is strong and constitutive and can cause high level of transgenic expression in leaves, fruits, tubers, roots and other organs (18). Kozak sequence was used to boost the expression of INFα2b gene (19). His tag sequence is for recognition and purification of the interested protein from other proteins of a transgenic plant and factor Xa is for separation of the His tag from the purified interferon protein (20). As a model plant, tobacco benefits from well-established gene transfer and regeneration methodologies, and also the availability of many robust expression cassettes for the control of transgene expression. The Agrobacterium-mediated transformation is a method for transferring target genes into plants. The cocultivation of leaf discs with Agrobacterium can produce tobacco transformants with high quality and fertility (12). Considering of these advantages of plant systems in comparison with other systems, production of INFα-2b in tobacco transgenic lines were done. Since no report of interferon alpha production in plants in Iran has been expressed yet, this research could create a field of producing this drug in tobacco, in Iran.

## References

[1] Schillberg S, Emans N, Fischer R (2002). Antibody molecular farming in plants and plant cells.. Phytochem Rev..

[2] Fischer R, Schillberg S (2004). Molecular farming: Plant-made pH pharmaceuticals and technical proteins..

[3] Chelbi-Alix MK, Wietzerbin J (2007). Interferon, a growing cytokine family: 50 years of interferon research.. Biochimie..

[4] Kurane I, Meager A, Ennis FA (1986). Induction of interferon alpha and gamma from human lymphocytes by dengue virus-infected cells.. J Gen Virol..

[5] Rubinstein M, Levy WP, Moschera JA, Lai CY, Hershberg RD, Bartlett RT (1981). Human leukocyte interferon: isolation and characterization of several molecular forms.. Arch Biochem Biophys..

[6] Lee SH, Kelley S, Chiu H, Stebbing N (1982). Stimulation of natural killer cell activity and inhibition of proliferation of various leukemic cells by purified human leukocyte interferon subtypes.. Cancer Res..

[7] Strander H, Mogensen KE, Cantell K (1975). Production of human lymphoblastoid interferon.. J Clin Microbiol..

[8] Attallah AM, Fleisher TA, Hu R, Abdel-Gaffar H, Ibrahim S, Metwali A (1987). Immunological activities of recombinant interferon-alpha 2 and its A fragment.. J Interferon Res..

[9] Sen GC, Lengyel P (1992). The interferon system. A bird’s eye view of its biochemistry.. J Biol Chem..

[10] Maeda S, McCandliss R, Gross M, Sloma A, Familletti PC, Tabor JM (1980). Construction and identification of bacterial plasmids containing nucleotide sequence for human leukocyte interferon.. Proc Natl Acad Sci U S A..

[11] Lund P, Dunsmuir P (1992). A plant signal sequence enhances the secretion of bacterial ChiA in transgenic tobacco.. Plant Mol Biol..

[12] Bergmann L (1960). Growth and division of single cells of higher plants in vitro.. J Gen Physiol..

[13] De La Riva GA, González-Cabrera J, Vázquez-Padrón R, Ayra-Pardo C (1998). Agrobacterium tumefaciens: a natural tool for plant transformation.. Electron J Biotechn..

[14] Sambrook J, Russell DW (2001). Molecular cloning: a laboratory manual..

[15] Murashige T, Skoog F (1962). A revised medium for rapid growth and bio assays with tobacco tissue cultures.. Physiol plantarum..

[16] Dellaporta SL, Wood J, Hicks JB (1983). A plant DNA minipreparation: version II.. Plant Molec Biol..

[17] Schillberg S, Fischer R, Emans N (2003). ‘Molecular farming’ of antibodies in plants.. Naturwissenschaften..

[18] Kumar G, Ganapathi T, Bapat V (2007). Production of hepatitis B surface antigen in recombinant plant systems: an update.. Biotechnol Prog..

[19] Rajabi Memari H, Ramanan RN, Ariff AB (2010). Comparison of expression systems for the production of human interferon-α2b.. Cent Eur J Biol..

[20] Leelavathi S, Reddy VS (2003). Chloroplast expression of His-tagged GUS-fusions: a general strategy to overproduce and purify foreign proteins using transplastomic plants as bioreactors.. Mol Breeding..

